# Association of *TLR8* and *TLR9* polymorphisms with tuberculosis in a Chinese Han population: a case-control study

**DOI:** 10.1186/s12879-018-3485-y

**Published:** 2018-11-13

**Authors:** Ming-Gui Wang, Miao-Miao Zhang, Yu Wang, Shou-Quan Wu, Meng Zhang, Jian-Qing He

**Affiliations:** Department of Respiratory and Critical Care Medicine, West China Hospital, Sichuan University, No. 37, Guo Xue Alley, Chengdu, 610041 Sichuan Province People’s Republic of China

**Keywords:** Toll-like receptors, Single nucleotide polymorphism, Tuberculosis

## Abstract

**Background:**

Toll-like receptor (*TLR*) single nucleotide polymorphisms (SNPs) have been associated with regulation of *TLR* expression and development of active tuberculosis (TB). The objectives of this study were to determine whether *TLR8* and *TLR9* SNPs were associated with the development of latent TB infection (LTBI) and the subsequent pulmonary TB (PTB) in a Chinese Han population.

**Methods:**

Two independent samples were enrolled. The first sample contained 584 TB cases and 608 controls; the second sample included 204 healthy controls, 201 LTBI subjects and 209 bacteria-confirmed active PTB patients. Three SNPs (rs3764880, rs187084 and rs5743836) were genotyped. The associations between the SNPs and risk of LTBI or PTB were investigated using unconditional logistic regression analysis.

**Results:**

The A-allele of *TLR8* rs3764880 SNP was protective against the development of TB in males (A vs G, OR = 0.58, 95%CI = 0.37–0.91). The AA genotype of rs3764880 SNP was found to increase the risk of PTB among females with an OR of 4.81 (1.11–20.85). The G allele of *TLR9* SNP rs187084 was found to increase the risk of PTB (G vs A, *P* = 0.01, OR = 1.48, 95% CI = 1.10–2.00), the significance was also observed under dominant genetic models. The GA-genotype of *TLR9* rs187084 SNP was found to increase the risk of PTB with an OR of 1.68 (1.07–2.65), but was found to decrease the risk of MTB infection with an OR = 0.64 (0.41–0.98). *TLR9*_rs5743836 SNP was excluded from the data analyses, because the minimum allele frequency was< 1%.

**Conclusions:**

Our findings in two independent samples indicated that SNPs in *TLR8* and *TLR9* were associated with the development of TB, and highlight that SNPs may have different effects on disease pathogenesis and progression.

## Background

Tuberculosis (TB), caused by infection with *Mycobacterium tuberculosis* (MTB), remains one of the world’s deadliest communicable diseases. According to the World Health Organization, an estimated 10.4 million people developed TB and 1.7 million died of the disease in 2016 [1]. However, most individuals exposed to MTB experience latent MTB infection (LTBI) and do not develop active disease. Considerable evidence suggests that host genetic factors play a key role in determining an individual’s susceptibility to TB [2–5].

Toll-like receptors (*TLRs*) are a class of pattern recognition molecules, which are known to play important roles in the innate and adaptive immune system [6], by recognizing pathogen-associated molecular patterns. Most *TLRs* are expressed on the cell surface, whereas other *TLRs* (3, 7, 8, and 9) are expressed intracellularly [7, 8]. *TLR7, TLR8*, and *TLR9* have been implicated in immune diseases due to their ability to recognize oligonucleotide-based (RNA-and DNA-based) molecular patterns as agonists [9].

*TLR8* is located in the membranes of the endosomal compartment and recognize single-stranded RNA, regulating in the induction of interferon (IFN) and inflammatory cytokines [10–12]. Previous studies have shown that *TLR8* variants influence the expression of *TLR8* [13–15]. The *TLR8* single nucleotide polymorphism (SNP) rs3764880 (Met1Val) regulates the translation of the two main *TLR8* isoforms, and plays a significant part in the immune response [12, 15]. A study conducted by Davila et al. [14], was the first to demonstrate that SNPs in *TLR8* were associated with TB in adults. Since *TLR8* is located on chromosome X (Xp22.3-p22.2), males carrying a single copy of the defective allele may have higher risk of TB. In addition, several studies have shown that the G allele of *TLR8* rs3764880 was associated with TB susceptibility in males [14, 16–18]. These studies demonstrated that the rs3764880 SNP in *TLR8* play important roles in TB.

The ligands for *TLR9* are DNA-containing CpG motifs [19]. *TLR9* is located in the endosomal compartment of plasmacytoid dendritic cells and monocytes/macrophages [19], and plays a vital role in autoimmune diseases and inflammatory diseases by the regulation of type I IFN and inflammatory cytokines [19–21]. The rs187084 and rs5743836 SNPs located in the promoter are the most important and have been associated with various inflammatory diseases [9, 21–24]. Previous functional analyses have shown that both rs187084 and rs5743836 SNPs influence the transcription of *TLR9* by regulation of promoter activity [22, 25, 26]. Previous studies indicated that *TLR9* is one of the most important receptors in the control of infections with pathogens such as hepatitis C virus [22], Brucella [27], and MTB [23, 28, 29]. Some studies found that the rs187084 in *TLR9* showed no association with TB in Vietnam and Iran [28, 30]. However, no study has explored the association between rs187084 and MTB infection or the process from LTBI to TB. The rs5743836 in *TLR9* showed a strong association with tuberculosis in African-Americans and Caucasians [31], while the association was not found in Vietnam [28] or Mexico population [29]. Wu L et al. [32] also reported that rs5743836 was a risk factor for LTBI.

These findings demonstrated that *TLR8* and *TLR9* play important roles in infectious diseases, and also emphasized the role of the rs3764880 SNP in *TLR8* and rs187084 and rs5743836 SNPs in *TLR9*. To date, the SNPs of *TLR8* and *TLR9* have been studied in association with susceptibility to TB, but such studies addressing host genetic susceptibility to TB was limited, whether an association implies susceptibility for developing active disease or just acquisition of MTB infection is unclear. In this study, we investigated the associations of SNPs of *TLR8* (rs3764880) and *TLR9* (rs187084, rs5743836) with TB in a Chinese Han sample. Then we explored the associations of these SNPs with LTBI or PTB in a second sample of Chinese Han individuals.

## Materials and methods

### Subjects

#### First sample

A cohort of 584 new TB patients above 15 years of age were recruited from the West China Hospital of Sichuan University. Diagnosis was based on the following criteria: culture positive and/or smear positive for MTB and/or histopathological findings of TB and clinical and/or radiographic presentation consistent with TB, with positive response to anti-TB therapy. 608 unrelated healthy controls (with unknown status of MTB infection), matched with cases by gender and age, were selected from individuals attending the outpatient department of the West China Hospital for annual physical examination. Healthy controls with a history of prior anti-TB treatment were excluded. All participants diagnosed with diabetes mellitus, human immunodeficiency virus (HIV) co-infection, or in receipt of immunosuppressive therapy, were excluded.

#### Second sample

A cohort of 614 participants were recruited: patients with pulmonary TB (PTB) (*n* = 209), LTBI subjects (*n* = 201), and healthy controls (HC) without MTB infection (*n* = 204). As with the first sample, the subjects in the second sample were recruited from the West China Hospital in Chengdu. Eligibility criteria for PTB patients included: 1) ≥ 18 years old; 2) diagnosis of PTB based on sputum smear examinations for acid-fast bacilli and/or the culture of MTB. Both LTBI and HC subjects were unrelated and asymptomatic contacts of bacteria-confirmed active TB patients. LTBI was defined as a positive result for the QuantiFERON-TB Gold In-Tube and HC was defined as a negative result for the same assay, and patients belonging to both subgroups had no radiological evidence of active TB, negative sputum smear and culture, and no history of TB. Exclusion criteria included a positive serological test for HIV infection, organ transplantation, primary immunodeficiency, cancer, and treatment with immunosuppressive drugs, endocrine disorders such as diabetes, autoimmune or chronic renal disease, and extra-pulmonary TB cases.

All protocols were approved by the ethics committee of the West China Hospital of Sichuan University. Written informed consent was obtained from all participants involved in this study. Demographic characteristics of all participants were collected from a detailed questionnaire.

### Genotyping

A volume of 2–5 ml blood samples were collected in EDTA tubes (BD Vacutainers, Franklin Lakes, NJ, USA) from each participant. Genomic DNA was extracted from whole blood following a protocol described elsewhere [33]. Three SNPs (rs3764880, rs187084, rs5743836) were genotyped using a custom-by-design 2 × 48-Plex SNPscan™ Kit (Cat#: G0104, Genesky Biotechnologies Inc., Shanghai, China) as described previously [34]. This kit was developed according to a patented SNP genotyping technology by Genesky Biotechnologies Inc., which was based on double ligation and multiplex fluorescence PCR. As a quality control measure, 5% of the samples were genotyped in duplicate using the same method to check for concordance. Assessment of genotypes was done by laboratory personnel without any prior knowledge of the diagnosis of the subjects.

### Statistical analyses

The clinical and demographic characteristics were compared using the student’s test or ANOVA for continuous variables and with the χ2 test or Fisher’s exact test for categorical variables. *P* <  0.05 was considered significant.

Hardy-Weinberg Equilibrium (HWE) in healthy controls was tested using asymptotic Pearson’s chi-square tests for each SNP, in each study. Because the *TLR8* rs3764880 SNP is located on chromosome X, we tested for HWE in females only. Since we used loose-matching to select cases and controls, associations between polymorphisms and risk of TB, LTBI or PTB were investigated using unconditional logistic regression analysis [35], adjusting for age and gender, and smoking as in the analysis of the first sample. Because this was an exploratory analysis, we did not introduce a correction for multiple comparisons. The results were expressed by odds ratios (ORs) and 95% confidence intervals (95% CIs). All statistical analyses were carried out using SPSS statistical software, release 19.0.

## Results

### Clinical information of study subjects

Demographic and clinical parameters of the patients are summarized in Table 1. As shown in Table 1, 584 TB cases and 608 controls from the Chinese Han population were enrolled in the first sample, with no significant difference in age and gender ratio. More smokers were found among the TB patients as compared with controls (*P* = 0.012). 614 eligible participants were enrolled in the second sample, including 209 PTB cases, 201 LTBI, and 204 HC (Table 1). There was no significant difference in gender among the three groups, while the mean age was significantly different among the PTB, LTBI and control groups (*P* <  0.001). No deviations from HWE were detected in all groups (*P* > 0.05). Genotyping data for the three SNPs were successfully obtained for ≥98.8% of the subjects. However, *TLR9*_rs5743836 SNP was excluded from the data analyses, because the minimum allele frequency (MAF) was ≤1%.

**Table 1:** Clinical characteristics of the study population

	First sample			Second sample			
	TB patients (584)	Control (608)	*P* value	PTB (209)	LTBI (201)	HC (204)	*P* value
Age (mean ± SD)	36.68 ± 15.61	37.18 ± 15.68	0.608	38.76 ± 16.97	49.09 ± 15.91	45.71 ± 14.90	< 0.001
Gender							
Males, n	299	302	0.598	107	95	93	0.503
Females, n	285	306		102	106	111	
Smokers, n(%)	173 (29.62%)	141 (23.19%)	0.012	–	–	–	–

*Abbreviations*: *TB* tuberculosis, *PTB* pulmonary tuberculosis, *LTBI* latent tuberculosis infection, *HC* healthy control

### *TLR8* rs3764880 and TB disease progression

The genotype and allele distributions of this SNP in the first sample are shown in Table 2. Firstly, a decreased frequency of the minor allele A of *TLR8*_rs3764880 was found in the male TB patient group, and found to be protected against TB (OR = 0.58, 95%CI = 0.37–0.91, *P* = 0.02) after adjusting for age and smoking in the first sample. No difference was found in the genotype frequencies among male patients in this sample. There was no difference in both allele and genotype frequencies among female subjects in the first sample. Further analysis was performed in the second sample (Table 3). No significant differences in the allele frequency of *TLR8*_rs3764880 SNP were observed for male or female subjects when comparing PTB disease with LTBI groups or when comparing LTBI group with HC subjects. In contrast, an increased frequency of the AA genotype of rs3764880 was observed in female patients, and found to increase the risk of PTB (OR = 4.81, 95%CI = 1.11–20.85, *P* = 0.04) when comparing PTB disease with LTBI groups. No differences in frequencies of *TLR8*_rs3764880 genotypes were observed for male or female patients when comparing LTBI group with HC subjects.

**Table 2:** The genotype and allele frequencies of rs3764880 in patients with TB and controls

Genotypes and allele frequencies	Cases, n (%)	Controls, n (%)	OR (95%CI)	*P* value
Males				
TLR8 rs3764880 major allele G	253 (86.6)	236 (79.5)	1	Reference
TLR8 rs3764880 minor allele A	39 (13.4)	61 (20.5)	0.58 (0.37–0.91)	0.02
Females				
TLR8 rs3764880 GG	203 (71.2)	209 (68.8)	1	Reference
TLR8 rs3764880 GA	76 (26.7)	82 (27.0)	0.93 (0.65–1.35)	0.71
TLR8 rs3764880 AA	6 (2.1)	13 (4.3)	0.49 (0.18–1.31)	0.16
TLR8 rs3764880 major allele G	482 (84.6)	500 (82.2)	1	Reference
TLR8 rs3764880 minor allele A	88 (15.4)	108 (17.8)	0.84 (0.61–1.14)	0.26

*Abbreviations*: *TB* tuberculosis, *OR* odds ratio, *CI* confidence interval. P value adjusted for age and smoking

**Table 3:** The genotype and allele frequencies of rs3764880 in the second sample

Genotypes and allele frequencies	Cases, n (%)	Controls, n (%)	OR (95%CI)	*P* value
PTB and LTBI				
Males				
TLR8 rs3764880 major allele G	84 (79.2)	84 (88.4)	1	Reference
TLR8 rs3764880 minor allele A	22 (20.8)	11 (11.6)	1.92 (0.85–4.32)	0.12
Females				
TLR8 rs3764880 GG	65 (63.7)	71 (67.6)	1	Reference
TLR8 rs3764880 GA	28 (27.5)	31 (29.5)	1.08 (0.56–2.09)	0.83
TLR8 rs3764880 AA	9 (8.8)	3 (2.9)	4.81 (1.11–20.85)	0.04
TLR8 rs3764880 major allele G	158 (77.5)	173 (82.4)	1	Reference
TLR8 rs3764880 minor allele A	46 (22.5)	37 (17.6)	1.56 (0.93–2.62)	0.09
LTBI and HC				
Males				
TLR8 rs3764880 major allele G	84 (88.4)	76 (82.6)	1	Reference
TLR8 rs3764880 minor allele A	11 (11.6)	16 (17.4)	0.53 (0.23–1.25)	0.15
Females				
TLR8 rs3764880 GG	71 (67.6)	70 (63.1)	1	Reference
TLR8 rs3764880 GA	31 (29.5)	36 (32.4)	0.85 (0.47–1.52)	0.58
TLR8 rs3764880 AA	3 (2.9)	5 (4.5)	0.64 (0.15–2.84)	0.56
TLR8 rs3764880 major allele G	173 (82.4)	176 (79.3)	1	Reference
TLR8 rs3764880 minor allele A	37 (17.6)	46 (20.7)	0.83 (0.51–1.34)	0.44

*Abbreviations*: *PTB* pulmonary tuberculosis, *LTBI* latent tuberculosis infection, *HC* healthy control, *OR* odds ratio, *CI* confidence interval. P value adjusted for age

#### *TLR9* rs187084 and TB disease progression

The genotype and allele distributions of this SNP in the first and second samples are shown in Table 4. Associations between *TLR9* rs187084 and the development of TB, PTB or LTBI were assessed in dominant, and recessive models. Firstly, analysis in the first sample detected no difference in allele and genotype frequencies among TB patients and controls. No significant differences were detected in *TLR9* rs187084 SNP between TB patients and controls under any genetic model in the first sample. To further explore whether this SNP was associated with susceptibility to PTB or MTB infection, the second sample was analyzed. When comparing PTB patients with LTBI group, significant associations with developing disease were observed after adjusting for age and gender (Table 4). The GA-genotype of *TLR9* rs187084 SNP was found more frequent in PTB cases, and was found to increase the risk of PTB with an OR of 1.68 (95% CI: 1.07–2.65). The prevalence of minor allele G of rs187084 was significantly higher in patients with PTB than that of controls (41.9% vs 32.3%, OR = 1.48, 95% CI = 1.10–2.00, *P* = 0.01). The significance was also observed under dominant genetic models (Table 4), indicating that the GG and GA genotypes increased risk susceptibility to develop active PTB from LTBI status. When comparing the LTBI group with HC subjects, a significant difference was found in genotype distributions. GA-genotype was found to decrease the risk of MTB infection (GA vs AA, *P* = 0.04, OR = 0.64, 95% CI = 0.41–0.98). However, no differences in allele distributions or genotype distributions under different genetic models were observed between LTBI group and HC subjects.

**Table 4:** Association between rs187084 and TB, LTBI and PTB susceptibility in Chinese Han population

	First sample				Second sample							
	TB, n(%)	Controls, n(%)	OR^a^ (95% CI)	P^a^	PTB, n(%)	LTBI, n (%)	OR^b^ (95% CI)	P^b^	LTBI, n(%)	HC, n (%)	OR^b^ (95% CI)	P^b^
Genotype												
GG	75 (12.9)	74 (12.3)	1.07 (0.74–1.55)	0.70	41 (19.6)	29 (14.5)	1.80 (0.99–3.27)	0.06	29 (14.5)	30 (14.8)	0.83 (0.46–1.52)	0.55
GA	267 (46.0)	271 (44.9)	1.06 (0.83–1.36)	0.64	93 (44.5)	71 (35.5)	1.68 (1.07–2.65)	0.03	71 (35.5)	93 (45.8)	0.64 (0.41–0.98)	0.04
AA	238(41.0)	259 (42.9)	1	Reference	75 (35.9)	100 (50.0)	1	Reference	100 (50.0)	80 (39.4)	1	Reference
Allele												
G	417 (35.9)	419 (34.7)	1.05 (0.88–1.24)	0.6	175 (41.9)	129 (32.3)	1.48(1.10–2.00)	0.01	129 (32.3)	153 (37.7)	0.82 (0.61–1.10)	0.19
A	743 (64.1)	789 (65.3)	1	Reference	243 (58.1)	271 (67.8)	1	Reference	271 (67.8)	253 (62.3)	1	Reference
Dominant												
GG + GA	342 (59.0)	345 (57.1)	1.07 (0.85–1.35)	0.59	134 (64.1)	100 (50.0)	1.70 (1.12–2.57)	0.01	100 (50.0)	123 (60.6)	0.68 (0.46–1.02)	0.06
AA	238 (41.0)	259 (42.9)	1	Reference	75 (35.9)	100 (50.0)	1	Reference	100 (50.0)	80 (39.4)	1	Reference
Recessive												
GG	75 (12.9)	74 (12.3)	1.05 (0.74–1.48)	0.8	41 (19.6)	29 (14.5)	1.40 (0.81–2.42)	0.22	29 (14.5)	30 (14.8)	1.03 (0.59–1.79)	0.93
GA + AA	505 (87.1)	530 (87.7)	1	Reference	168 (80.4)	171 (85.5)	1	Reference	171 (85.5)	173 (85.2)	1	Reference

*Abbreviations*: *TB* tuberculosis, *PTB* pulmonary tuberculosis, *LTBI* latent tuberculosis infection, *HC* healthy control, ^a^, adjusted for age, gender and smoking with logistic regression; ^b^, adjusted for age and gender with logistic regression; OR, odds ratio; CI, confidence interval

## Discussion

Multiple groups have investigated associations between the three SNPs which we examined in the in current study and TB susceptibility in a variety of populations, but the results were inconsistent [14, 17, 18, 28, 30, 32, 36–40]. Few studies have classified LTBI and HC without MTB infection. The current study first analyzed the association between *TLR8/9* SNPs and TB in the first sample, and then further explored the *TLR8/9* variants with LTBI and active PTB in the second sample. Our results suggest that genetic variants might have different roles in the development of active PTB and LTBI.

Davila et al. [14] for the first time found that four SNPs (rs3764879G/C, rs3788935G/A, rs3761624G/A, rs3764880G/A) in *TLR8* showed evidence of association with TB susceptibility with minor alleles showing an increased susceptibility to PTB in males in Russian and Indonesian populations. Furthermore, associations have also been found in Turkish male children [17], Pakistan population [41], and South African population [18]. However, neither Kobayashi et al. [42] nor Chimusa et al. [43] showed any association between rs3764880 and TB susceptibility. Our results showed that the rs3764880 A allele in *TLR8* greatly reduced TB risk in males. This result was consistent with that in a Pakistan population [41], which showed the rs3764880 A allele had a protective role against TB,but different from that in Turkish [17], South African [18], Russian and Indonesian populations [14], which showed the rs3764880 A allele increased susceptibility to TB in males. Subsequently, our data in the second sample provided new evidence for our understanding of the role of rs3764880 in the development of LTBI and PTB. The data suggested that the AA-genotype of rs3764880 increased susceptibility to PTB among females, however, might decrease susceptibility to LTBI. The difference between our results and that of other studies may be due to differences in race, or living environment. Therefore, the detection of this SNP among LTBI subjects may provide important information in the assessment of their risk profiles for susceptibility to development of PTB. Previous studies have found that the SNPs in *TLR8* might have gender effects across the genetic association studies on TB susceptibility [14, 17, 18, 41]. Our results also found a gender difference: male carriers of rs3764880 allele A showed a decreased risk for TB, and females carrying the AA genotype increased the risk of PTB when compared with LTBI. What’s more, this gender effect was both demonstrated in the two independent study samples.

Several investigators have studied the roles of *TLR9* SNPs in TB. As reported previously in studies of Vietnam and Iran [28, 30], we found the rs187084 in *TLR9* showed no association with TB in the first sample. In the second sample, the G-allele of *TLR9* rs187084 greatly increased PTB risk, and this association was also observed under a dominant genetic model, which suggested a risk role of the allele G. However, the GA genotype decreased the risk of MTB infection. A study by Digna Rosa Velez et al. [31] showed a strong association between rs5743836 and tuberculosis in African-Americans and Caucasians population, while the association was not found in Vietnam [28] or Mexico population [29]. Another study conducted by Wu L et al. [32] showed that rs5743836 was a risk factor for LTBI and its MAF was 0.27 in a Chinese population in Shanghai city. The MAF of this SNP is high among African-Americans, Africans and Caucasians [31], while low in the Mexican population [29] and Vietnamese people [28]. These studies, thus indicated that the MAF of rs5743836 may vary between different ethnic groups. In this study, we found the MAF of rs5743836 was less than 1%. Since our sample size may not be large enough for a SNP with low MAF, and may potentially lead to false associations with the phenotype investigated, the rs5743836 was excluded in the data analyses. The MAF of rs5743836 in Beijing Han population from the International HapMap Project (http://www.1000genomes.org/) is less than 0.01, which support our result and indicates a low mutation rate in Chinese Han population. Therefore, the higher rs5743836 MAF in the study of Wu L et al. may suggest that populations other than Chinese Han were included in the study [32].

It has been discussed that some SNPs may actually be associated with MTB infection [36, 37], some may be associated with active TB [38, 39] and other studies suggested some SNPs may have different impacts on the susceptibility to LTBI and PTB [32, 40, 44]. Lu et al. found that an immunity-related GTPase family M SNP was associated with active TB and LTBI, but also plays opposite roles in the development of active TB and LTBI [44]. Our results provide strong evidence that the allele “A” of the polymorphism rs3764880 was associated with decreased risk of tuberculosis in the first sample. The genotype “AA” of rs3764880 was more commonly found in female PTB patients compared with LTBI, indicating this genotype increases the possibility of progression of tuberculosis infection to disease in the second sample. For the polymorphism of rs187084 in *TLR9*, we found the genotype “GA” and allele “G” was more common in PTB patients compared with LTBI, which demonstrated that this allele increased the risk of progression from tuberculosis infection to active disease. However, the GA-genotype was found to reduce the risk of MTB infection, thereby indicating that the results of our two samples were inconsistent and may have been caused by a number of factors. It may be caused by the following reasons. First, we included all TB patients in our first sample without differentiating between the different types of TB, while we only included PTB patients in the second sample. Genetic polymorphisms may have different effects on PTB and extra-pulmonary TB (EPTB), which may be due to the fact that different immune mechanisms are involved in PTB and EPTB [45]. Second, the results in the first sample may be explained, when the distinction between LTBI and HC without the infection of MTB is ignored. In other words, the results may be different because of the differences within the control group. Therefore, it is reasonable that these SNPs showed different results in the two samples.

Our study faced some intrinsic limitations. First, small sample size in the second sample may have limited our ability to detect potential influence of *TLR* SNPs on the susceptibility to both LTBI and PTB. Further studies are therefore necessary to validate these associations in larger sample sizes and other populations. Second, TB case of the first sample included both clinical diagnosed and bacteria confirmed TB patients, regardless of their types, which could potentially reduce the validity of our conclusions. Third, LTBI and HC subjects included in the second sample were asymptomatic contacts of bacteria-confirmed active TB patients, the assessment of exposure to an active TB case relied on self-reported behavior, which may not be accurate among participants and may cause misclassification bias (once exposed, those controls could become cases). Forth, it is likely that linkage disequilibrium patterns differed among other populations and the Han Chinese population in this study, and thus, those associations may not replicate exactly [14, 28, 31]. A plausible explanation is that the SNPs that we identified are in linkage disequilibrium with another mutation that either confers a functional or a regulatory change within those genes. Hence, to interpret results across studies, future experiments should cover all common variation in the associated *TLR8/9* gene region in order to pinpoint the causal polymorphism. Furthermore, one potential limitation in our study was a lack of correction for multiple comparisons. The reported statistically significant results would need to be regarded as nominally significant.

## Conclusion

Our study provides another evidence supporting the importance of host genetic variability in TB susceptibility. We identified the allele A of the rs3764880 that appears protective against TB among males of Chinese Han ancestry. Our results also highlight that polymorphisms may have different effects on disease pathogenesis and progression. This study may help to identify those TB-affected individuals most susceptible to disease, and to improve our understanding of the effects of genetic heterogeneity on the development of the different stages of TB.

## Abbreviations

CIs: Confidence intervals
EPTB: extra-pulmonary tuberculosis
HC: Healthy controls
HIV: Human immunodeficiency virus
HWE: Hardy-Weinberg Equilibrium
IFN: Interferon
LTBI: Latent Mycobacterium tuberculosis infection
MAF: Minimum allele frequency
MTB: Mycobacterium tuberculosis
ORs: Odds ratios
PTB: Pulmonary Tuberculosis
SNP: Single nucleotide polymorphism
TB: Tuberculosis
TLRs: Toll-like receptors
